# Micro-CT Features of Lung Consolidation, Collagen Deposition and Inflammation in Experimental RSV Infection Are Aggravated in the Absence of Nrf2

**DOI:** 10.3390/v15051191

**Published:** 2023-05-18

**Authors:** Teodora Ivanciuc, Igor Patrikeev, Yue Qu, Massoud Motamedi, Yava Jones-Hall, Antonella Casola, Roberto P. Garofalo

**Affiliations:** 1 Department of Pediatrics, University of Texas Medical Branch, Galveston, TX 77555, USA; teivanci@utmb.edu (T.I.); yuqu@utmb.edu (Y.Q.); ancasola@utmb.edu (A.C.); 2 Department of Ophthalmology & Visual Sciences, University of Texas Medical Branch, Galveston, TX 77555, USA; igpatrik@utmb.edu (I.P.); mmotamed@utmb.edu (M.M.); 3 Biomedical Engineering Center, University of Texas Medical Branch, Galveston, TX 77555, USA; 4 Department of Veterinary Pathobiology, Texas A&M College of Veterinary Medicine and Biomedical Sciences, College Station, TX 77843, USA; yavajh@cvm.tamu.edu; 5 Department of Microbiology and Immunology, University of Texas Medical Branch, Galveston, TX 77555, USA; 6 Sealy Institute for Vaccine Sciences, University of Texas Medical Branch, Galveston, TX 77555, USA

**Keywords:** respiratory syncytial virus, Nrf2, collagen, micro-CT, airway remodeling

## Abstract

Severe respiratory syncytial virus (RSV) infections in early life have been linked to the development of chronic airway disease. RSV triggers the production of reactive oxygen species (ROS), which contributes to inflammation and enhanced clinical disease. NF-E2-related factor 2 (Nrf2) is an important redox-responsive protein that helps to protect cells and whole organisms from oxidative stress and injury. The role of Nrf2 in the context of viral-mediated chronic lung injury is not known. Herein, we show that RSV experimental infection of adult Nrf2-deficient BALB/c mice (*Nrf2^−/−^*; Nrf2 KO) is characterized by enhanced disease, increased inflammatory cell recruitment to the bronchoalveolar compartment and a more robust upregulation of innate and inflammatory genes and proteins, compared to wild-type Nrf2^+/+^ competent mice (WT). These events that occur at very early time points lead to increased peak RSV replication in Nrf2 KO compared to WT mice (day 5). To evaluate longitudinal changes in the lung architecture, mice were scanned weekly via high-resolution micro-computed tomography (micro-CT) imaging up to 28 days after initial viral inoculation. Based on micro-CT qualitative 2D imaging and quantitative reconstructed histogram-based analysis of lung volume and density, we found that RSV-infected Nrf2 KO mice developed significantly greater and prolonged fibrosis compared to WT mice. The results of this study underscore the critical role of Nrf2-mediated protection from oxidative injury, not only in the acute pathogenesis of RSV infection but also in the long-term consequences of chronic airway injury.

## 1. Introduction

NF-E2-related factor 2 (Nrf2) is an evolutionary conserved redox-responsive protein that helps to protect cells and whole organisms from oxidative stress and injury [1]. It is estimated that Nrf2 regulates a network of hundreds of antioxidant and anti-inflammatory genes [2]. The transcription factor Nrf2, a basic leucine zipper (bZIP) protein, contains seven functional NRF2-ECH homology (Neh) domains, known as Neh1-Neh7, with Neh2 considered the major regulatory domain. Neh2 is located at the N terminus of Nrf2 and interacts with cytoplasmic Kelch-like ECH-associated protein 1 (Keap1), a component of the Cullin-3-based E3 ubiquitin ligase complex. In unstressed cells, the Keap1 forms a complex in the cytoplasm with Nrf2 and binds to the ETGE and DLG motifs on the Neh2 domain of Nrf2, which brings Nrf2 into the Keap1-Cul3-E3-ubiquitin ligase complex. Oxidative stress or reactive electrophiles can induce conformational changes in this complex and disrupt the Nrf2-Keap1 binding domain [3]. This complex dissociates, and then free Nrf2 translocates to the nucleus and binds to the antioxidant-responsive element (ARE) sequences of AOE genes to promote gene transcription [4]. Using *Nrf2*-knockout (Nrf2 KO) murine models in different organ models and under different conditions of stimulation/stress, Nrf2 has been shown to regulate a variety of target genes, such as antioxidant genes, xenobiotic-metabolizing enzymes, many of which have been traditionally classified as part of the phase II detoxification system, glutathione homeostasis, solute channels, proteome maintenance and innate immune responses (reviewed in [2]). On the other hand, disruption of *Keap1* (KO mice) leads to enhanced nuclear accumulation of Nrf2 and elevated expression of Nrf2-regulated genes [5].

In the respiratory system, the Nrf2 response has been shown, among others, to be critical for protection against pulmonary inflammation, asthma, hyperoxia and acute lung injury (reviewed in [6,7]). A decline in the Nrf2 pathway is associated with severe chronic obstructive pulmonary disease (COPD) [6], and in experimental animal models, Nrf2 has been shown to be involved in tissue protection against the development of fibrosis and collagen deposition [8,9]. In a mouse model of bleomycin (BLM)-induced lung fibrosis, it was shown that compared to wild-type mice, Nrf2 knockout mice (KO) exhibited increased lung weight, inflammation, hydroxyproline content and fibrotic score [10]. Treatment with sulforaphane (SFN), an Nrf2 activator, in a pulmonary fibrosis mouse model, attenuated alveolitis, fibrosis, apoptosis and lung oxidative stress by increasing the expression of antioxidant enzymes, including NAPDH, Nqo1, Ho1, superoxide dismutase and catalase [11]. Similar data have been reported in mouse models of radiation-induced lung injury [12].

While some viral infections have been shown to activate Nrf2, among them hepatitis B and C viruses, human cytomegalovirus, Kaposi’s sarcoma-associated herpes virus and Marburg virus [13,14,15,16,17,18], respiratory viruses, including RSV, hMPV, influenza and SARS-CoV-2 are associated with a progressive reduction in Nrf2 cellular levels and subsequent inhibition of AOE expression [7,19,20]. Indeed, the Nrf2 pathway has been shown to play a protective role in the murine airways against RSV-induced acute lung injury and oxidative stress: more severe RSV disease, including higher peak viral titers, augmented inflammation and enhanced disease were found in Nrf2 KO mice compared to Nrf2-competent mice [19,21]. Since these studies focused only on the acute manifestations of RSV infection, antiviral response and airway inflammation, the role of Nrf2 in protection from viral-mediated chronic airway disease, structural tissue alteration and features of airway fibrosis are not known. Thus, the current study was designed to investigate both aspects of early response to viral infection as well as progressive anatomical changes in the lung architecture using high-resolution micro-computed tomography (micro-CT) imaging in response to RSV infection in Nrf2 KO mice compared to Nrf2-competent WT mice. Changes in lung morphology/density were reconstructed in a group of RSV-infected mice at 7, 14, 21 and 28 days based on micro-CT images. Overall, the results of this study demonstrate, for the first time, that Nrf2 plays a protective role in RSV-induced chronic lung alterations and identified micro-CT as a sensitive imaging tool to study lung structural changes using mouse models of respiratory viral infection.

## 2. Materials and Methods

### 2.1. Preparation of Virus Stock

Respiratory long-strain syncytial virus was grown in HEp-2 cells (American Type Culture Collection, Manassas, VA, USA) and purified, as described elsewhere [22]. A methylcellulose plaque assay was used to determine the virus titer and ranged from eight to nine log10 plaque-forming units (PFUs)/mL.

### 2.2. Mice and Infection Protocol

*Nrf2*^−/−^ (Nrf2 KO) mice on a mixed C57BL/6 and AKR background were generated as previously described and received as a generous gift from Drs. Jefferson Chan at the University of California San Francisco and Karen T. Liby at Michigan State University, East Lansing, Michigan. These mice were backcrossed onto a BALB/c background for eight generations and were found to be 99% congenic (analysis performed by Jackson Laboratory, Bar Harbor, ME), as previously described [23]. Experiments were performed using 21-week-old Nrf2 KO and wild-type (WT) female mice. Three to five female mice per each group were used in our studies for each assay performed. Under light anesthesia of ketamine and xylazine, mice were infected intranasally (i.n.) with 50 µL of RSV diluted in phosphate-buffered saline (PBS) at a dose of 5 × 10^6^ plaque-forming units (PFUs) or PBS (control). Weight loss and illness scores were recorded daily throughout the course of infection [24]. At indicated time points, randomly assigned animals were euthanized, and bronchoalveolar lavage fluid (BALF) was collected for quantification of cytokines/chemokines and type I interferons measurements, and lungs tissue was collected for viral titration and pulmonary histopathology [24]. High-resolution micro-computed tomography (micro-CT) imaging was used to investigate the progressive anatomical changes in the lungs.

### 2.3. BALF Cells, Bioplex Analysis and Virus Titration

BALF from each group was collected at day 1 post-infection, and the total number of cells was determined using trypan blue. Cytopsin preparations and measurements of 23 cytokines/chemokines and type I interferons alpha and beta in BAL fluid were performed as previously described [25,26]. At day 5 post-infection, RSV-infected mice were sacrificed, and the lungs were harvested for virus quantification [24].

### 2.4. RNA Isolation and Gene Expression Profiling

Total RNA was isolated from each individual PBS- or RSV-infected lung with RNeasy Mini Kit followed by DNase I treatment (Qiagen) per the manufacturer’s recommendations. RNA samples were quantified using a Nanodrop Spectrophotometer (Nanodrop Technologies, Wilmington, DE, USA). The quality of the total RNA was confirmed via the 260/280 nm ratio, which varied from 1.9 to 2.0. Synthesis of cDNA was performed with 4 μg of total RNA in a 20 μL reaction volume using the reagents from iScript^TM^ Advanced cDNA Synthesis kit for real-time (RT)-quantitative (q) PCR (Bio-Rad Laboratories, Hercules, CA, USA), according to manufacturer’s instructions. cDNA was mixed with SsoAdvanced Universal SYBR Green Supermix and Precision Blue^TM^ Real-Time PCR Dye, and 10 μL of reaction mixture was added to each well of the inflammatory cytokines and receptors M384 PCR Array (Bio-Rad Laboratories, Hercules, CA, USA) to determine changes in messenger RNA (mRNA) levels. The qPCR was performed using a CFX384 Touch Real-Time PCR Detection System (Bio-Rad). The following housekeeping genes were used as internal controls and validated in CFX Maestro software: glyceraldehyde-3-phosphate dehydrogenase (*Gapdh*), hypoxanthine phosphoribosyltransferase (Hprt), TATA-box binding protein (*Tbp*), glucuronidase beta (*Gusb*) and heat shock protein 90 alpha (cytosolic), class B member 1 (*Hsp90ab1*). The normalization and all the data analysis were performed according to the manufacturer’s instructions using Bio-Rad CFX Maestro software package. The complete list of genes is provided in the Supplementary Material, Table S1.

### 2.5. In Vivo Micro-CT Imaging and Analysis

Micro-CT imaging was performed to evaluate the presence of remodeling/fibrosis, as previously described [27,28]. Groups of PBS and RSV-infected Nrf2 KO and WT mice were scanned serially at 7, 14, 21 and 28 days after inoculation. Under light anesthesia (ketamine and xylazine), mice were scanned while spontaneously breathing. Duration of the procedure for each mouse was approximately 2 min. Mouse whole lung images were acquired using a Quantum GX2, PerkinElmer (Waltham, MA, USA) micro-CT small animal scanner, with the following parameters for images acquisition: X-ray tube voltage 70 KV, X-ray tube current 80 µA and 140 µm isotropic reconstructed voxel size. Mice that had spontaneous deviations from the regular breathing patterns, as evidenced by lack of image visual clarity, e.g., due to swallows or irregular breathing movements, were omitted from analyses or rescanned.

### 2.6. Image Post-Processing: Lung Segmentation Protocols and Analysis

For each acquisition, a stack of 512 × 512 × 371 cross-sectional images stored in unsigned 16-bit file format was produced. The Siemens Image Research Workplace 4.2 (for image post-processing) software was used to analyze the reconstructed datasets. The micro-CT images were converted to Hounsfield Units (HUs), with settings for the density of air at −1000 HU and density of water at 0 HU. The following outcome measures were determined as the cross-sectional area: total lung volume, tissue density histograms and average density [29].

### 2.7. Lung Histology

At day 28 post-infection, all mice underwent the last micro-CT and then were sacrificed, and lung samples were collected for histopathology. Formalin-fixed lungs were paraffin-embedded, cut into 5 μm sections and stained for collagen using Masson’s Trichrome. The slides were analyzed by a trained experimental veterinary pathologist with expertise in mouse lungs. Slides were scanned using Aperio ImageScope Digital slide scanner (Leica Biosystems, Wetzlar, Germany). The Visiopharm^®^ Software (Version 2022.01) was used to analyze the images. The Visiopharm APP custom alghoritm was employed for the quantification of collagen deposition, expressed as percentage of blue staining in the lung slides. The collagen ratio was determined by dividing the area of collagen staining by the total tissue area evaluated (region of interest). This number was multiplied by 100 to determine the percent collagen per tissue [30,31,32].

### 2.8. Statistical Analysis

The data were analyzed via one-way ANOVA and two-tailed unpaired Student’s *t*-test for samples with unequal variances using GraphPad Prism (Version 5.02; GraphPad Software, Inc., San Diego, CA, USA). Data are presented as mean  ±  SEM. *p* value less than 0.05 value was considered significant.

### 2.9. Ethics Statement

All procedures involving mice in this study were performed in accordance with the recommendations in the Guide for the Care and Use of Laboratory Animals of the National Institutes of Health. The Institutional Animal Care and Use Committee (IACUC) of the University of Texas Medical Branch at Galveston approved the animal study protocol (IACUC #9001002) used in these studies.

## 3. Results

### 3.1. Enhanced Disease and Viral Replication in Absence of Nrf2

**Figure 1:**
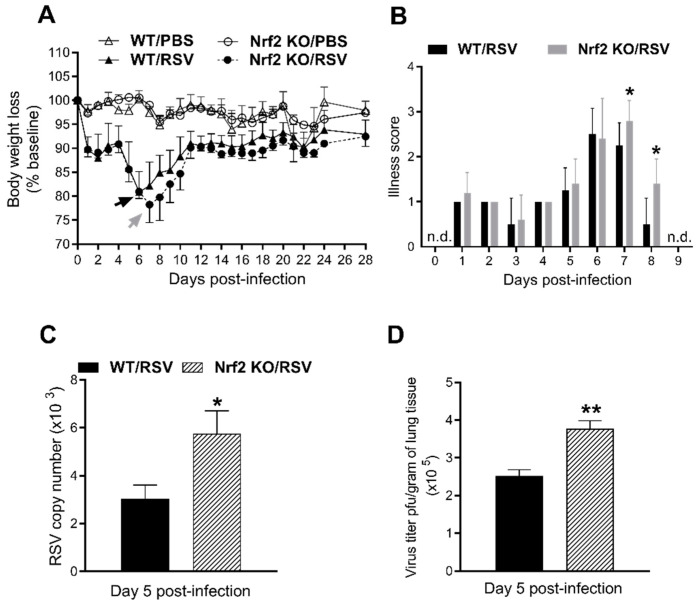
Disease and lung viral replication in the absence of Nrf2. (**A**) Body weight and (**B**) illness scores. Data are shown as percent change in body weight relative to the starting weight on day 0 (mean ± SEM). Arrows represent the day of peak weight loss for RSV/WT (black arrow, day 6 pi) and RSV/Nrf2 KO (gray arrow, day 7 pi). On day 5 pi, lungs were isolated from infected mice and viral load was determined via qRT-PCR (**C**) and plaque assays (**D**). Data represent the mean  ±  SEM (*n*  =  3–5 mice/group). * *p* < 0.05, ** *p*  <  0.01 vs. RSV/WT at day 5 post-infection. n.d.= not detected.

Groups of Nrf2 KO and WT control were infected intranasally with 5 × 10^6^ PFUs of RSV or inoculated with PBS. Clinical disease was determined via assessment of daily body weight (loss) and a standardized illness score (Figure 1A,B). PBS-inoculated mice from both groups did not exhibit signs of illness at any time point after infection, showing that the Nrf2 absence gene does not lead to disease. RSV infection in mice produced a bimodal weight loss pattern, with an early phase (first 4 days post-infection) of ~9–11% of their initial weight followed by a second peak of weight loss. Following RSV infection, WT mice reached a maximum % weight loss of 19% ± 4% at day 6 post-infection, while Nrf2 KO mice reached 22% ± 4% at day 7 (Figure 1A). Body weight for both RSV-infected groups increased slightly over the next days, recovering ~93% for WT and ~92% for Nrf2 of their initial weight by day 28. As shown in Figure 1B, Nrf2 KO infected mice showed a significant higher illness score at the peak of their body weight loss compared with infected WT ones (* *p* < 0.05). To determine peak viral load in the lung, mice were sacrificed on day 5 after infection, and total lung tissue was collected for qPCR and plaque assays. As shown in Figure 1C, infected Nrf2 KO mice had significantly higher RSV genome copy numbers compared to WT animals (* *p* < 0.05). Similar results were found for the plaque assays of lung homogenates (** *p* < 0.01, Figure 1D).

### 3.2. BALF Cellularity and Cytokines Are Increased at Early Time Points in Absence of Nrf2

**Figure 2:**
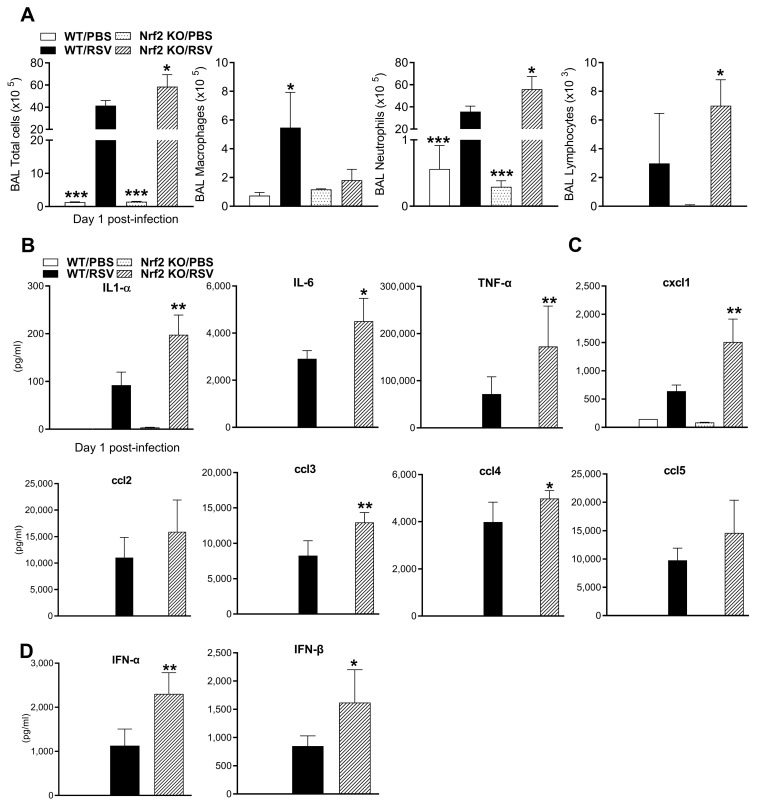
BALF cells and cytokines after RSV infection in the absence of Nrf2. (**A**) Total cells and macrophage, neutrophil and lymphocyte numbers in BALF of WT and Nrf2 KO mice (day 1 pi). Levels of cytokines (**B**), chemokines (**C**), and type I IFN (**D**) in BAL expressed as pg/mL. Data represent the mean  ±  SEM (*n*  =  4 mice/group). * *p* < 0.05, ** *p*  <  0.01, *** *p* < 0.001 when compared with WT/RSV at day 1 post-infection.

Groups of RSV-infected or PBS-inoculated mice were sacrificed on day 1 post-infection (pi) to collect BALF for total and differential cell counts and for the analysis of cytokines and chemokines. Total cellularity in the BALF of PBS-inoculated mice was not significantly different between WT and Nrf2 KO mice (Figure 2A). As expected, RSV infection was associated with an increase in BALF total cell counts and neutrophil numbers compared to PBS mice (*** *p* < 0.001, Figure 2A). Compared to RSV-infected WT mice, RSV-infected Nrf2 KO mice had a significantly greater number of total BAL cells and neutrophils (Figure 2A). The number of lymphocytes was higher while macrophages were lower in RSV-infected Nrf2 KO compared to RSV-infected control mice.

Cytokines and chemokines in BAF samples were measured using a multi-plex cytokine array. As shown Figure 2B, the concentrations of IL-1α, IL-6 and TNF-α were significantly greater in RSV-infected Nrf2 KO mice compared with infected WT mice. Concentrations of the chemokines ccl3 (MIP-1α) and ccl4 (MIP-1β) reached statistical significance in BALF samples of RSV-infected Nrf2 KO (Figure 2C). Consistent with neutrophil numbers in BALF, the concentration of the neutrophil chemoattractant cxcl1 (KC) was significantly increased in RSV-infected Nrf2-deficient mice compared to infected WT mice (Figure 2C). Albeit not statistically significant, infected Nrf2 KO mice had greater levels of ccl2 (MCP-1) and ccl5 (RANTES) compared to infected WT mice. Other cytokines/chemokines that were measured with the 23-plex were not statistically different between infected Nrf2 KO and WT mice. RSV infection in Nrf2 KO mice was also associated with significantly greater levels of both IFN-α and -β, measured via ELISA in BALF samples, compared with infected WT mice (* *p* < 0.05, ** *p* < 0.01, Figure 2D).

### 3.3. Inflammatory and Immunoregulatory Cytokine Gene Expression in the Lung

To determine a broader spectrum of RSV-regulated innate genes in the lung in the absence of Nrf2, we employed a multi-target PCR array to compare cytokine and cytokine receptor mRNA levels. The complete list of 90 genes in the array is provided in the Supplementary Material (Table S1). To minimize the noise in the data, we set a threshold for gene selection at a 1.5-fold increase or decrease (fold change, fc) in signal intensity and a *p*-value < 0.05, in RSV-infected mice compared to their respective PBS-inoculated controls. Overall, RSV-infected Nrf2 KO had 43 upregulated and 12 downregulated genes, while RSV-infected WT mice had 44 upregulated and 12 downregulated genes identified in the lungs (Table 1). Among those upregulated by RSV infection, the expression levels were significantly higher in Nrf2 KO mice compared to WT mice for (C-C motif) ligand 20 (*Ccl20*, *14.83-fold in KO* vs. *WT*), (C-X-C motif) ligand *Cxcl1 (4.07-fold)*, *Oncostatin M* (*Osm, 3.88 fold*)), interleukin (*Il)1b (3.76-fold)*, interleukin receptor antagonist (*Il1rn*, *3.46-fold)*, *Ccl4* (3.46-fold), *Ccl2* (3.43-fold), *Ccl7* (3.19-fold), *Ccl3* (2.59-fold), *Tnf* (2.59-fold), *Ccl12* (2.5-fold), *Csf3* (2.32-fold) and *Il1a* (2.24-fold). Some genes, including *Ccl8*, *Cxcl5*, interleukin *Il11* and *Il13*, were significantly upregulated only in RSV-infected Nrf2 KO mice, and others, including (C-C motif) receptor 8 (*Ccr8*), *Il17f*, interleukin 2 receptor beta (*Il2rb*), tumor necrosis factor (ligand) superfamily member 10 (*Tnfsf10*) and *Tnfsf4,* were significantly upregulated only in RSV-infected WT mice. The mRNA data corroborated, to a large extent, the results of cytokines measured at the protein level in BAL fluids (Figure 2B,C).

The genes that were significantly downregulated in Nrf2 and WT mice following RSV infection compared to PBS are shown in Table 2.

### 3.4. Longitudinal Assessment of Infected Mouse Lung via Micro-CT Imaging, 3D Reconstruction and Quantitative Analysis

**Figure 3:**
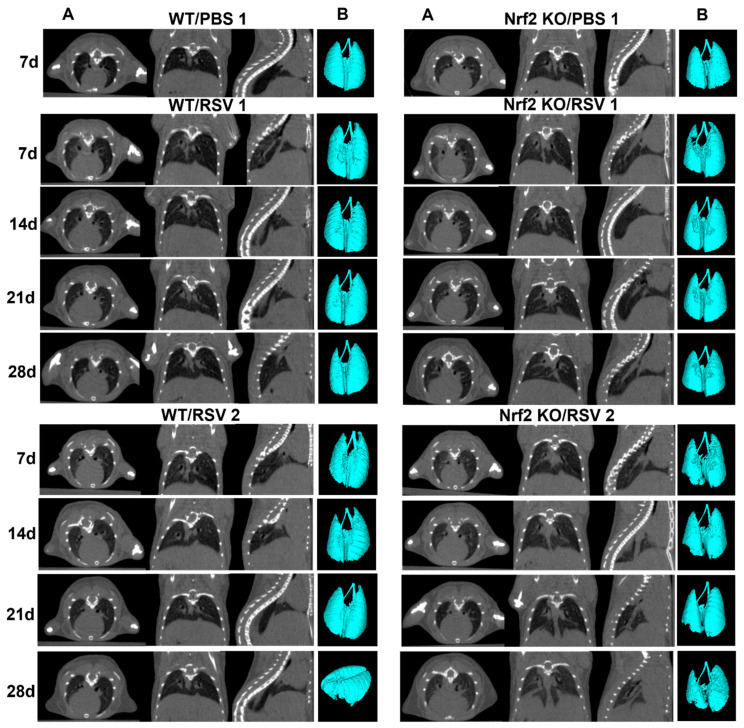
Longitudinal *in vivo* lung micro-CT images after RSV infection. Nrf2 KO and WT female mice were inoculated i.n. with RSV at dose 5 × 10^6^ PFU or PBS. (**A**) Lung micro-CT images of a representative PBS, and two RSV-infected WT mice (left panel) and two Nrf2 KO mice (right panel) take at the indicated time points after infection. Multiplanar views (axial, dorsal, sagittal) at the level of T6 thoracic vertebrae. Micro-CT images shown for PBS mice only on day 7 after infection. (**B**) Representative 3D reconstructions of PBS and RSV-infected WT and Nrf2 KO mice from the images obtained *in vivo* (Hounsfield units (HUs) HU: (−1000, −400)). *n* = 3–5 mice/group.

**Figure 4:**
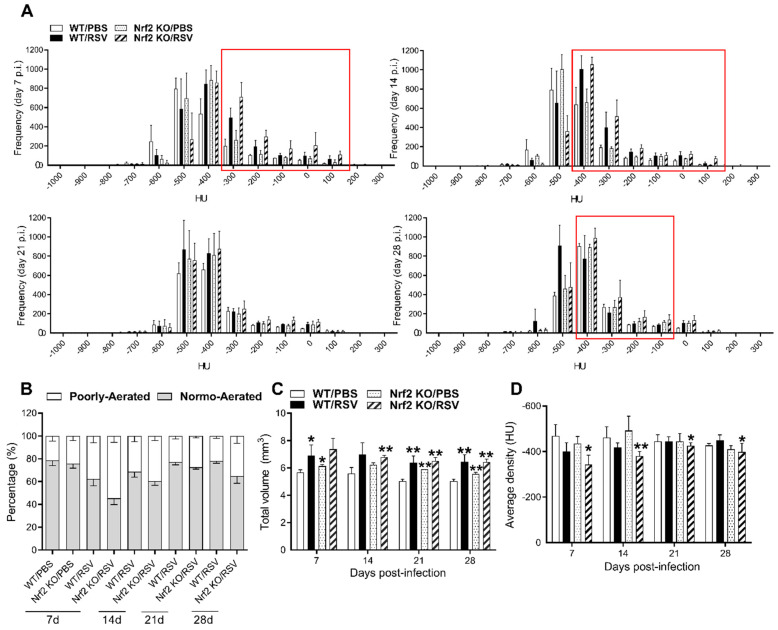
Quantitative analysis of lung micro-CT imaging. (**A**) CT histograms of lungs over time. Mean frequency histogram of the number of pixels having a particular Hounsfield unit (HU). The extracted data are from the whole lung segmentation for each timepoint per animal/group. Changes in frequency numbers in the lung between RSV-infected Nrf2 KO and WT mice and compared with PBS controls (red quadrant). HU values on the *x*-axis and frequency on the *y*-axis (**B**) Lung aeration degrees expressed as percentage of normo- and poorly-aerated tissues at 7, 14, 21 and 28 days for RSV-infected Nrf2 KO and WT animals. Data for PBS Nrf2 KO and WT-inoculated mice are shown on day 7 only. Normally aerated ((−900, −400) HU) and poorly aerated ((−399, 100) HU) regions. Increase in total lung volume (**C**) and average lung density (**D**) in RSV-infected Nrf2 KO and WT mice vs. PBS controls. All data are expressed as mean  ±  SEM (*n* = 3–5 mice/group). * *p* < 0.05, WT/RSV vs. WT/PBS, Nrf2 KO/RSV vs. Nrf2 KO/PBS at day 7 p.i.; ** *p* < 0.01 WT/RSV vs. WT/PBS, Nrf2 KO/RSV vs. Nrf2 KO/PBS at days 14, 21, and 28 p.i. WT/RSV vs. Nrf2 KO/RSV at days 14 and 28 post-infection.

To determine if a lack of Nrf2 leads to time-dependent changes in the lung parenchyma (such as patchy density and fibrosis) following RSV infection, we used *in vivo* high-resolution micro-CT to compare four groups of mice: RSV-infected WT and Nrf2 KO mice, and their PBS control mice with repeated imaging performed on days 7, 14, 21 and 28. Representation of lung micro-CT images, quantification of histograms, lung volume and lung densities are shown in Figure 3 and Figure 4. Multiplanar images were acquired from anesthetized mice over regular breathing patterns. Figure 3A shows representative CT scans of two RSV-infected WT, two RSV-infected Nrf2 KO mice and two PBS mice, visualizing longitudinal lung images over the four-week period following RSV infection. In the axial views, the cross-sectional area at the level of the sixth thoracic vertebra was used for comparison between groups. Features of lung consolidation were observed throughout the left lungs of RSV-infected mice in both groups of infected mice compared to PBS controls. However, RSV-infected KO Nrf2 mice showed more areas of segmental consolidation. The three-dimensional (3D) reconstruction images (Figure 3B) provide additional visualization of the changes in the lung, including a loss of air space in RSV-infected compared to PBS-treated mice at each time point of observation.

Computed CT scan images of all animals in each group and for each time point were used to generate the histograms shown in Figure 4. Each pixel in the CT images has a value that can be mapped to the density of the tissue being imaged. This scale is referred to as CT number or Hounsfield units (HUs), which is based on water being zero (0 HU) and air (−1000 HU). To isolate regions of interest (ROIs) in the lung, micro-CT images were segmented at the sixth thoracic vertebra, and within the segmented region, the frequency of pixels in the density interval of −1000 to 200 HU was determined. This interval was divided into 100 bins, and the data were plotted as frequency of occurrence (expressed as the number of pixels) at each density bin. As shown in Figure 4A, histograms of the extracted data displayed a greater number of pixels (i.e., tissue-denser areas), represented by an increase in Hounsfield unit (HU) in the lung ROIs of RSV-infected WT and Nrf2 KO mice, compared with their PBS-treated controls (Figure 4A). For quantitative assessment of the lung lesions, HU ranges were applied to semi-automatically segmented lung distinguishing in normally aerated ((−900, −400) HU) and poorly aerated ((−399, 100) HU) regions. Longitudinal quantification of lung aeration degrees confirmed the increase in the percentage of poorly aerated tissue following RSV infection, particularly more prominent in the group of Nrf2 KO mice (Figure 4B). The peak of poorly aerated lung regions occurred on day 7 but could be observed also at later time points up to day 28 in the infected Nrf2 KO group. Moreover, as shown in Figure 4C, a comprehensive analysis of all micro-CT images of mouse lungs demonstrates that RSV infection resulted in a highly significant increase in total lung volumes as early as day 7 and remained increased for up to day 28 compared with their PBS controls. RSV infection resulted in a significant increase in mean lung density in Nrf2 KO mice for all time points of observation (* *p* < 0.05 on days 7, 21 and 28 pi, and ** *p* < 0.01 on day 14 pi, Figure 4D). Compared to PBS controls, RSV-infected WT mice displayed an increase in lung densities only on days 7 and 14 post-infection (Figure 4D). Collectively, these data indicated that RSV infection in Nrf2-deficient mice produced remodeling in the lung, as evidenced by micro-CT images and consistent with lung tissue of higher density and total volume, which is indicative of collagen deposition and increased lung rigidity.

### 3.5. Histochemical Analysis of Collagen Deposition

**Figure 5:**
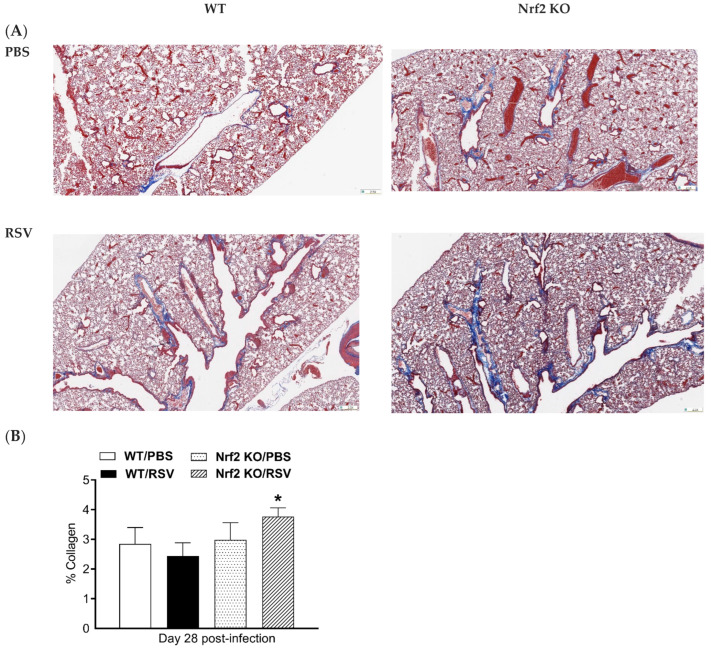
Masson’s trichrome staining for collagen in lung sections. (**A**) Representative images of Masson trichrome-stained lung sections of PBS-treated (top) and RSV-infected (bottom) WT and Nrf2 KO mice at day 28 p.i. Scale bars, 200 μm at ×2.5 magnification. (**B**) Collagen content percentage. The percentage of stained area was assessed with Visiopharm^®^ Software (Version 2022.01). Data are expressed as mean  ±  SEM (*n* = 3–5 mice/group). * *p* < 0.05.

On day 28 post-infection, mice were sacrificed after acquiring the micro-CT images, and lungs were fixed with 10% (*v*/*v*) neutral-buffered formalin and embedded in paraffin. For histological examination, 4 µm sections of fixed embedded tissues were cut, and Masson’s trichrome (MT) staining was used to quantify collagen content. Representative images of MT-stained lung sections are shown in Figure 5A. Collagen deposition was most prominent expanding the space around large airways (bronchi) and large vessels (arteries and veins). However, collagen was also noted with the alveolar septa and in the perivascular space of small blood vessels. Quantitative analysis of collagen content in each mouse group indicates that only the RSV-infected Nrf2 KO mice had a significantly higher % of MT staining compared to their control PBS mice on day 28 (Figure 5B).

## 4. Discussion

Our studies using an Nrf2 KO mouse model on a BALB/c background show that Nrf2 plays an important role in both early (hrs and days) cellular, inflammatory and antiviral responses as well as prolonged (up to 4 weeks) alterations in lung tissue. Increased numbers of neutrophils and lymphocytes were identified in BALF one day after viral inoculation in Nrf2 KO compared to control WT mice, as we previously reported in Nrf2 KO mice on a BL6 background [24]. Similar to the other Nrf2 KO models, we also found an increased viral load in the lung compared to Nrf2-competent controls [24,33]. Using protein arrays and focused mRNA analysis of cytokine/chemokine genes, we expanded our understanding of the Nrf2-regulated innate immune response beyond the AOE gene network, which is triggered by RSV infection of the lung. We found several cytokines and chemokines, both at the protein and mRNA level that were induced at higher levels in RSV-infected Nrf2 KO mice compared to WT mice. These results extend our previous observations in a BL6 Nrf2 KO mouse model [24] and confirm that Nrf2 plays a central role in the modulation of antiviral and inflammatory gene responses to RSV. Interestingly, we found that the most upregulated gene in infected Nrf2 KO mice compared to infected WT was *ccl20* (Table 1). CCl20 and its receptor, CCR6, control the migration of dendritic cells and Th17 CD4^+^ T cells to the site of infection and inflammation [34,35]. In the context of RSV infection, it was reported that antibody neutralization of CCL20 protein or using mice deficient in CCR6 results in decreased lung pathology and more efficient viral clearance [36]. Our results, herein, and previous work from us and others have shown that increased lung pathology and viral replication are features of the lack of Nrf2 in mouse models [24,33]. Whether the CCL20-CCR6 axis plays a role in the features of lung pathology and viral replication observed in Nrf-2 KO mice remains to be determined (see below).

Various mechanisms may explain how Nrf2 deficiency leads to increased expression of several cytokines and chemokines (Figure 2 and Table 1). Nrf2, for example, inhibits the NF-κB transcription factor, which plays a key role in regulating the expression of several RSV-induced genes in the lung. In previous studies, we directly showed greater nuclear translocation of RelA in the lung in the absence of Nrf2 [24]. Another mechanism could be that Nrf2 suppresses macrophage inflammatory responses by blocking specific inflammatory cytokine production. In this regard, we found that alveolar macrophage (AM) depletion from mouse lung prior to RSV inoculation leads to the disruption of key inflammatory mediators, including IL-6, TNF-α and IFNs-α/β production [37]. There is further evidence that Nrf2 interferes with the induction of IL-6 and IL-1β genes in lipopolysaccharide (LPS)-stimulated macrophages by binding in the proximity of their promotor region, blocking the recruitment of RNA polymerase II [38]. Moreover, the data showing that RSV-infected Nrf2 KO mice had greater levels if type I IFN in the BALF (Figure 1) confirm our previous observations [24]. Indeed, Nrf2 has been shown to function as a negative regulator of the adaptor molecule STING upstream of the signaling cascade that leads to IFN and antiviral gene expression in response to viral infection [39,40]. Removing Nrf2 would then result in a more robust IFN/antiviral gene response to viruses. However, not surprisingly, based on the complex nature of interaction between RSV and type I IFNs, increased peak RSV titers in the lung of Nrf2 KO mice occurred despite elevated levels of INF-α/β compared to WT mice.

The role of Nrf2 in the replication of RNA viruses in the lung has been confirmed in this study with Nrf2 KO mice on a BALB/c background (Figure 1C,D). Similar to our findings in this work, adult Nrf2 KO mice on the ICR background [33] or older mice on a BL6 background were shown to have increased RSV and hMPV peak replication and shedding [24]. We also found increased SARS-CoV-2 replication in BALB/c Nrf2 KO mice compared to WT controls [20]. We proposed several different possibilities to explain such findings, including: (1) A relative defect in the antioxidant defense system and enhanced oxidative response in the absence of Nrf2, supported by evidence that exogenous treatment of cells or mice with antioxidant enzymes or synthetic compounds with antioxidant activity reduces the replication/viral load [25,41,42]. (2) Nrf2 has been shown to alter T-helper cell 1/Th2 balance, and the oxidative stress might lead to a loss of naïve T cells and a decrease in Th1-mediated immunity [43]. In addition, as mentioned previously, we found that CCL20 is strongly upregulated in infected Nrf2 KO mice, potentially affecting antiviral response and leading to increased RSV replication [36]. (3) We discovered antiviral properties of the cellular endogenous H_2_S pathway [26] and a relationship between Nrf2 and the H_2_S-generating enzymes, as suggested by the significant reduction in the expression of CSE, CBS and 3-MST, which we observed in RSV-infected Nrf2 KO mice [24].

In addition to altered early inflammatory responses and innate antiviral immunity, genetic deficiency of Nrf2 was associated with more severe and long-lasting lung damage and fibrosis in RSV-infected mice. These lung pathological alterations were identified using highly sensitive imaging technology along with morphologic and quantitative analysis that were used for the first time in our study of experimental RSV infection. Specifically, micro-CT histogram-based analysis, total lung volume and density changes in the lung were used in our study. The histogram-based analysis showed that in RSV-infected Nrf2 KO mice, all three time points (7, 14 and 28 pi) produced plots, which were shifted rightward towards the denser region with an increase in the number of pixels compared to their PBS control and compared to WT-infected animals (Figure 4A). In the case of RSV-infected WT mice, this changes in the density profiles that were shifted to the denser region only on days 7 and 14 post-infection when compared with their PBS control. Similarly, while RSV-infected WT mice showed an increase in poorly aerated lung tissue 7 and 14 days after infection, infected Nrf2 KO mice had a significantly higher percent of poorly aerated tissue as early as day 7 pi compared to Nrf2-competent WT controls. This indicator of lung disease was still altered at later time points (day 14 and 28) in Nrf2 KO mice only (Figure 4B). Using other micro-CT-derived parameters to longitudinally follow the different groups of mice, we found that both RSV-infected Nrf2 KO and WT mice had significantly higher total lung volume with respect to their control mice as early as day 7 pi and up to day 28. Moreover, RSV infection resulted in a significant increase in mean lung density at all time points of observation, only in Nrf2 KO mice. In agreement with micro-CT data, MT staining of lung sections on day 28 pi showed increased deposition of collagen only in RSV-infected Nrf2 KO.

Several considerations should be considered in interpreting these data. First, evidence that RSV experimental infection alone in adult mice leads to airway fibrosis/remodeling has not been previously demonstrated. Using a neonatal model of infection followed by reinfection one month later, Kimura et al. found that these mice developed peribronchial and perivascular inflammation and fibrosis one week after reinfection, compared with the sham-infected control mice [44]. In studies by Kellar et al., juvenile C57BL6 mice (3-week-old) but not adult mice (8-week-old) exhibited a distinct myeloid recruitment pattern in response to RSV infection, αSma expression (indicative of myofibroblast activity) and increased hyaluronan deposition in the lung parenchyma (72 h post-infection). As noted, both these studies had a short period from infection to collection of the lung and, therefore, it is not possible to conclude that the observed features of fibrosis were indeed sustained. Nonetheless, our micro-CT studies in BALB/c WT mice support the findings that lung density and volume are increased at least up to 14 days after infection. The second consideration is that studies showing more robust lung fibrosis in experimental RSV infection have been performed using a co-exposure model with an allergen or a profibrotic agent. Indeed, histological analyses of MT-stained lung sections from 3-week-old mice exposed to ovalbumin and then RSV displayed collagen deposition, which is indicative of airway remodeling [45]. Wang et al. demonstrated that RSV administration resulted in increased collagen type-1 deposition in the lung tissues of an animal model bleomycin-induced pulmonary fibrosis [46]. In very elegant studies, Tian et al. [29] repetitively administrated (15 times) Toll-like receptor 3 (TLR3) agonist poly(I:C) to adult BAL/c mice, resulting in enhanced fibrosis observed via micro-CT, as in our study, and via immunohistochemistry. Viral antigen/replications and histopathology assessment of cellular inflammation (beyond day 5), which could contribute to lung fibrosis, as captured using micro-CT imaging, were not addressed in this study. Published data in a mouse model of RSV infection up to 150 days showed airway obstruction and chronic inflammation present for several weeks, with no evidence of viral replication beyond day 7 [47]. However, staining for collagen was not performed. Thus, the contribution of inflammatory cells to the process of collagen deposition and other features of pulmonary remodeling in RSV-infected mice will need to be further investigated. Nonetheless, other studies by our group in human lung epithelial cells infected with RSV and in mice infected with Sendai virus have shown that pneumoviruses induce mucosal growth factor response, EMT and the indicators of ECM remodeling, which persists after viral clearance and in the absence of obvious chronic inflammatory cells [48].

The third consideration is the role that Nrf2 plays in protecting the airways from a viral-mediated injury that can progress towards chronic aspects of tissue remodeling. Our data, herein, are the first to demonstrate that Nrf2 deficiency is clearly associated with enhanced chronic lung disease following a single acute viral infection. In previous work, Cho et al. showed that RSV-induced exacerbation of collagen accumulation was heightened in young adult Nrf2 KO mice that were exposed to hyperoxia as neonates [9]. The mechanism(s) of airway injury and lung fibrosis and the protective function of Nrf2 have been extensively studied in cellular and animal models (reviewed in [7,49]). Redox equilibrium, which is critically controlled by Nrf2, is central to several important processes in the lung and, therefore, an imbalance of oxidants and antioxidants as a consequence of RSV infection can trigger signaling pathways, transcription factors, immune responses, growth factors, etc., involved in the progression of collagen deposition and remodeling, all of which are further exacerbated by Nrf2 deficiency. We have previously shown that RSV infection activates a redox-sensitive signaling pathway that controls cytokine and chemokine gene expression and virus-induced lung inflammation and damage [50,51,52]. As such, antioxidant treatment of RSV-infected mice protected mice from clinical disease and improved lung function [25,50]. Moreover, we showed that RSV infection induced a significant decrease in the levels of AOEs and activity in epithelial cells [21] in the lung of infected mice and in children naturally infected with this respiratory virus [19]. These studies in mice have been mostly limited to the first week post-viral infection and have shown an overall replenishment of the AOE expression in the lung as the infection is terminated [19]. This may explain our findings herein with, mostly, a peak in lung alterations via micro-CT around 14 days in WT mice. On the other hand, under conditions of Nrf2 genetic deficiency and steady impaired AOE activity, RSV infection induced greater and more prolonged lung damage with fibrosis.

The relevance of these experimental observations for naturally acquired RSV infections in children and their potential to trigger long-term consequences such as airway remodeling will need further investigations. It is conceivable that genetic and environmental cofactors affecting the redox balance in the lungs may play a critical role, not only in the severity of acute disease but also in the chronic lung evolution of RSV infections. These may include, for example, genetic polymorphisms in the Nrf2 and/or AOE pathway [53] or in the innate inflammatory pathways triggered by RSV [54,55] or exposure to prooxidant agents such as tobacco smoke in the form of secondhand exposure [56].

## Figures and Tables

**Table 1:** Inflammatory cytokine ligand and receptor gene expression increased in the lung following RSV infection of wild-type WT and Nrf2 KO mice.

Genotype	Gene Symbol		Gene Name
WT	Nrf2 KO		
* FC	* FC		
5.69	5.68	*B2m*	Beta-2 microglobulin
6.92	3.55	*Ccl1*	Chemokine (C-C motif) ligand 1
2.17	2.70	*Ccl11*	Chemokine (C-C motif) ligand 11
21.15	52.90	*Ccl12*	Chemokine (C-C motif) ligand 12
2.77	2.65	*Ccl17*	Chemokine (C-C motif) ligand 17
5.74	5.98	*Ccl19*	Chemokine (C-C motif) ligand 19
193.79	664.12	*Ccl2*	Chemokine (C-C motif) ligand 2
7.62	112.99	*Ccl20*	Chemokine (C-C motif) ligand 20
5.84	1.84	*Ccl22*	Chemokine (C-C motif) ligand 22
172.11	445.40	*Ccl3*	Chemokine (C-C motif) ligand 3
282.90	977.64	*Ccl4*	Chemokine (C-C motif) ligand 4
7.48	13.70	*Ccl5*	Chemokine (C-C motif) ligand 5
97.70	311.62	*Ccl7*	Chemokine (C-C motif) ligand 7
9.50	12.49	*Ccr1*	Chemokine (C-C motif) receptor 1
2.20	1.75	*Ccr2*	Chemokine (C-C motif) receptor 2
5.53	7.15	*Ccr5*	Chemokine (C-C motif) receptor 5
11.93	11.42	*Csf1*	Colony stimulating factor 1 (macrophage)
15.95	14.83	*Csf2*	Colony stimulating factor 2 (macrophage)
166.25	384.91	*Csf3*	Colony stimulating factor 3 (macrophage)
21.35	86.81	*Cxcl1*	Chemokine (C-X-C motif) ligand 1
1862.03	3375.38	*Cxcl10*	Chemokine (C-X-C motif) ligand 10
16.79	28.27	*Cxcl13*	Chemokine (C-X-C motif) ligand 13
1532.46	1648.33	*Cxcl9*	Chemokine (C-X-C motif) ligand 9
6.07	7.29	*Cxcr2*	Chemokine (C-X-C motif) receptor 2
2.65	3.23	*Fasl*	Fas ligand (TNF superfamily, member 6)
10.66	12.69	*Ifng*	Interferon gamma
6.69	8.00	*Il15*	Interleukin 15
16.87	37.80	*Il1a*	Interleukin 1 alpha
16.44	61.88	*Il1b*	Interleukin 1 beta
43.84	151.66	*Il1rn*	Interleukin 1 receptor antagonist
62.00	78.08	*Il27*	Interleukin 27
2.72	2.16	*Il2rg*	Interleukin 2 receptor, gamma chain
5.22	5.53	*Il10ra*	Interleukin 10 receptor, alpha
1.84	1.78	*Il10rb*	Interleukin 10 receptor, beta
3.59	2.86	*Lta*	Lymphotoxin A
6.51	6.55	*Nampt*	Nicotinamide phosphoribosyl transferase
8.75	33.94	*Osm*	Oncostatin M
15.49	40.16	*Tnf*	Tumor necrosis factor
2.27	1.95	*Tnfrsf11b*	Tumor necrosis factor receptor superfamily, member 11b (osteoprotegerin)
2.97	n.s.	*Ccr8*	Chemokine (C-C motif) receptor 8
3.21	n.s	*Il17f*	Interleukin 17f
2.16	n.s.	*Il2rb*	Interleukin 2 receptor, beta
3.15	n.s.	*Tnfsf10*	Tumor necrosis factor (ligand) superfamily, member 10
3.08	n.s.	*Tnfsf4*	Tumor necrosis factor (ligand) superfamily, member 4
n.s.	3.62	*Ccl8*	Chemokine (C-C motif) ligand 8
n.s.	13.1	*Cxcl5*	Chemokine (C-X-C motif) ligand 5
n.s.	2.5	*Il11*	Interleukin 11
n.s.	7.88	*Il13*	Interleukin 13

* Fold change (FC) via RSV vs. PBS in each genotype. Criteria of statistical significance where the FC is greater than 1.5-fold with a *p* value ≤  0.05. n.s. not significant.

**Table 2:** Inflammatory cytokine ligand and receptor gene expression decreased in the lung following RSV infection of wild-type WT and Nrf2 KO mice.

Genotype	Gene Symbol		Gene Name
WT	Nrf2 KO		
* FC	* FC		
−1.90	−2.57	*Ccl6*	Chemokine (C-C motif) ligand 6
−2.37	−7.01	*Ccr3*	Chemokine (C-C motif) receptor 3
−1.67	−2.54	*Cxcl12*	Chemokine (C-X-C motif) ligand 12
−7.26	−3.55	*Cxcl15*	Chemokine (C-X-C motif) ligand 15
−2.71	−14.30	*IL5ra*	Interleukin 5 receptor, alpha
−3.75	−4.38	*Il16*	Interleukin 16
−7.72	−2.56	*Spp1*	Secreted phosphoprotein 1
−3.68	n.s.	*Ccr6*	Chemokine (C-C motif) receptor 6
−2.01	n.s.	*Ccr10*	Chemokine (C-C motif) receptor 10
−4.24	n.s.	*Il17b*	Interleukin 17B
−3.81	n.s.	*Tnfsf11*	Tumor necrosis factor (ligand) superfamily, member 11
−1.73	n.s.	*Il33*	Interleukin 33
n.s.	−3.14	*Cx3cl1*	Chemokine (C-X3-C motif) ligand 1
n.s.	−5.01	*Il5*	Interleukin 5
n.s.	−2.19	*IL4*	Interleukin 4
n.s.	−3	*Bmp2*	Bone morphogenetic protein 2
n.s.	−1.62	*Vegfa*	Vascular endothelial growth factor A

* Fold change (FC) by RSV vs. PBS in each genotype. Criteria of statistical significance where the FC is greater than 1.5-fold with a *p* value ≤  0.05. n.s. not significant.

## Data Availability

Not applicable.

## Supplementary Materials

The following supporting information can be downloaded at: https://www.mdpi.com/article/10.3390/v15051191/s1, Table S1: List of inflammatory cytokine and receptor mouse genes (total 90 genes).
