# Supplementary material for: Micro-CT Features of Lung Consolidation, Collagen Deposition and Inflammation in Experimental RSV Infection Are Aggravated in the Absence of Nrf2
Source: Viruses. 2023 May 18;15(5):1191. doi: 10.3390/v15051191 (PMC10223011; doi:10.3390/v15051191)
Supplement: Supplementary file 1 [file viruses-15-01191-s001.zip › viruses-2390686-supplementary.pdf]

Supplementary Table S1. List of inflammatory cytokines and receptors mouse genes (total 90 genes).

Gene Symbol	Gene name
Actb	Actin, beta
Aimp1	Aminoacyl tRNA synthetase complex-interacting
B2m	Beta-2 microglobulin
Bmp2	Bone morphogenetic protein 2
Ccl1	Chemokine (C-C motif) ligand 1
Ccl11	Chemokine (C-C motif) ligand 11
Ccl12	Chemokine (C-C motif) ligand 12
Ccl17	Chemokine (C-C motif) ligand 17
Ccl19	Chemokine (C-C motif) ligand 19
Ccl2	Chemokine (C-C motif) ligand 2
Ccl20	Chemokine (C-C motif) ligand 20
Ccl22	Chemokine (C-C motif) ligand 22
Ccl24	Chemokine (C-C motif) ligand 24
Ccl3	Chemokine (C-C motif) ligand 3
Ccl4	Chemokine (C-C motif) ligand 4
Ccl5	Chemokine (C-C motif) ligand 5
Ccl6	Chemokine (C-C motif) ligand 6
Ccl7	Chemokine (C-C motif) ligand 7
Ccl8	Chemokine (C-C motif) ligand 8
Ccl9	Chemokine (C-C motif) ligand 9
Ccr1	Chemokine (C-C motif) receptor 1
Ccr10	Chemokine (C-C motif) receptor 10
Ccr2	Chemokine (C-C motif) receptor 2
Ccr3	Chemokine (C-C motif) receptor 3
Ccr4	Chemokine (C-C motif) receptor 4
Ccr5	Chemokine (C-C motif) receptor 5
Ccr6	Chemokine (C-C motif) receptor 6
Ccr8	Chemokine (C-C motif) receptor 8
Cd40lg	CD40 ligand
Csf1	Colony stimulating factor 1 (macrophage)
Csf2	Colony stimulating factor 2 (granulocyte macrophage)
Csf3	Colony stimulating factor 3 (granulocyte)
Cx3cl1	Chemokine (C-X3-C motif) ligand 1
Cxcl1	Chemokine (C-X-C motif) ligand 1
Cxcl10	Chemokine (C-X-C motif) ligand 10
Cxcl12	Chemokine (C-X-C motif) ligand 12
Cxcl13	Chemokine (C-X-C motif) ligand 13
Cxcl15	Chemokine (C-X-C motif) ligand 15
Cxcl5	Chemokine (C-X-C motif) ligand 5
Cxcl9	Chemokine (C-X-C motif) ligand 9

Cxcr2	Chemokine (C-X-C motif) receptor 2
Cxcr3	Chemokine (C-X-C motif) receptor 3
Cxcr5	Chemokine (C-X-C motif) receptor 5
Fasl	Fas ligand (TNF superfamily, member 6)
Gapdh	Glyceraldehyde-3-phosphate dehydrogenase
Gusb	Glucuronidase, beta
Hprt	Hypoxanthine guanine phosphoribosyl transferase
Hsp90ab1	Heat shock protein 90 alpha (cytosolic), class B member 1
Ifng	Interferon gamma
Il10ra	Interleukin 10 receptor, alpha
Il10rb	Interleukin 10 receptor, beta
Il11	Interleukin 11
Il13	Interleukin 13
Il15	Interleukin 15
Il16	Interleukin 16
Il17a	Interleukin 17A
Il17b	Interleukin 17B
Il17f	Interleukin 17F
Il1a	Interleukin 1 alpha
Il1b	Interleukin 1 beta
Il1r1	Interleukin 1 receptor, type I
Il1rn	Interleukin 1 receptor antagonist
Il21	Interleukin 21
Il27	Interleukin 27
Il2rb	Interleukin 2 receptor, beta chain
Il2rg	Interleukin 2 receptor, gamma chain
Il3	Interleukin 3
Il33	Interleukin 33
Il4	Interleukin 4
Il5	Interleukin 5
Il5ra	Interleukin 5 receptor, alpha
Il6ra	Interleukin 6 receptor, alpha
Il6st	Interleukin 6 signal transducer
Il7	Interleukin 7
Lta	Lymphotoxin A
Ltb	Lymphotoxin B
Mif	Macrophage migration inhibitory factor
Nampt	Nicotinamide phosphoribosyltransferase
Osm	Oncostatin M
Pf4	Platelet factor 4
Spp1	Secreted phosphoprotein 1
Tbp	TATA box binding protein

Tnf	Tumor necrosis factor
Tnfrsf11b	Tumor necrosis factor receptor superfamily, member 11b
Tnfsf10	Tumor necrosis factor (ligand) superfamily, member 10
Tnfsf11	Tumor necrosis factor (ligand) superfamily, member 11
Tnfsf13	Tumor necrosis factor (ligand) superfamily, member 13
Tnfsf13b	Tumor necrosis factor (ligand) superfamily, member 13b
Tnfsf4	Tumor necrosis factor (ligand) superfamily, member 4
Vegfa	Vascular endothelial growth factor A
